# Biocrust reduces the soil erodibility of coral calcareous sand by regulating microbial community and extracellular polymeric substances on tropical coral island, South China Sea

**DOI:** 10.3389/fmicb.2023.1283073

**Published:** 2023-12-13

**Authors:** Lin Wang, Yu Huang, Qingsong Yang, Zhimao Mai, Feiyang Xie, Lina Lyu, Si Zhang, Jie Li

**Affiliations:** ^1^CAS Key Laboratory of Tropical Marine Bio-resources and Ecology, South China Sea Institute of Oceanology, Chinese Academy of Sciences, Guangzhou, China; ^2^University of Chinese Academy of Sciences, Beijing, China; ^3^Southern Marine Science and Engineering Guangdong Laboratory (Guangzhou), Guangzhou, China

**Keywords:** tropical coral island, biological soil crust, microbial community, extracellular polymeric substances, soil erodibility factor, soil nutrients

## Abstract

Tropical coral islands assume a pivotal role in the conservation of oceanic ecosystem biodiversity. However, their distinctive environmental attributes and limited vegetation render them highly susceptible to soil erosion. The biological soil crust (biocrust), owing to its significant ecological role in soil stabilization and erosion prevention, is deemed an effective means of mitigating soil erosion on coral island. However, existing research on the mechanisms through which biocrusts resist soil erosion has predominantly concentrated on arid and semi-arid regions. Consequently, this study will specifically delve into elucidating the erosion-resistant mechanisms of biocrusts in tropical coral island environments, South China Sea. Specifically, we collected 16 samples of biocrusts and bare soil from Meiji Island. High-throughput amplicon sequencing was executed to analyze the microbial community, including bacteria, fungi, and archaea. Additionally, quantitative PCR was utilized to assess the abundance of the bacterial 16S rRNA, fungal ITS, archaeal 16S rRNA, and cyanobacterial 16S rRNA genes within these samples. Physicochemical measurements and assessments of extracellular polymeric substances (EPSs) were conducted to characterize the soil properties. The study reported a significantly decreased soil erodibility factor after biocrust formation. Compared to bare soil, soil erodibility factor decreased from 0.280 to 0.190 t h MJ^−1^ mm^−1^ in the biocrusts. Mechanistically, we measured the microbial EPS contents and revealed a negative correlation between EPS and soil erodibility factor. Consistent with increased EPS, the abundance of bacteria, fungi, archaea, and cyanobacteria were also detected significantly increased with biocrust formation. Correlation analysis detected Cyanobacteria, Chloroflexi, Deinococcota, and Crenarchaeota as potential microbials promoting EPSs and reducing soil erosion. Together, our study presents the evidence that biocrust from tropical coral island in the South China Sea promotes resistance to soil erosion, pinpointing key EPSs-producing microbials against soil erosion. The findings would provide insights for island soil restoration.

## Introduction

Tropical coral islands hold a pivotal role in upholding the biodiversity of the oceanic ecosystem, as they serve as crucial waystations for migratory avifauna and marine mammals. Furthermore, they make significant contributions to the preservation of freshwater reserves and local climate regulation (Blackburn et al., 2004; Whittaker and Fernández-Palacios, 2007; Li et al., 2021). Coral calcareous sand stands as the predominant constituent of these islands; however, it poses challenges to the spontaneous growth of vegetation. Furthermore, the vegetation found on tropical coral islands is highly susceptible to degradation and notoriously challenging to restore once disrupted (Prabakaran and Paramasivam, 2014; Wolfe et al., 2015; Zong et al., 2021). This predicament arises due to a combination of distinctive environmental attributes, including a scarcity of soil clay and essential nutrients, elevated levels of salinity and alkalinity, intense heat and solar radiation, as well as recurrent periods of drought (Zhang et al., 2019). Without ecological function of vegetations, coral islands are highly prone to soil erosion. Therefore, erosion prevention and control are urgent issues that need to be addressed in the region currently. The employment of biological soil crusts (biocrusts) as a strategy to mitigate soil erosion on tropical coral islands bears resemblance to their efficacious implementation in desert ecosystems and other extreme habitats (Weber et al., 2016).

Biocrusts constitute a significant assemblage of soil particles and organisms, encompassing different species of cyanobacteria, microalgae, bacteria, microfungi, lichens, and bryophytes (Maier et al., 2018; Wang et al., 2022). Biocrusts thrive within the uppermost layer of the soil and serve as prominent biotic constituents within arid regions (Rodríguez-Caballero et al., 2022). According to the research by Rodriguez-Caballero et al. (2018), biocrusts approximately span 12% of the Earth’s surface area. As multifunctional communities, biocrusts could impact energy cycling, water retention, biogeochemical fluxes on a global scale, with crucial impacts on soil fertility, regional hydrology, and the soil’s resilience against erosive forces (Chamizo et al., 2012, 2016; Belnap et al., 2014; Couradeau et al., 2016; Mogul et al., 2017; Eldridge et al., 2020; Rodríguez-Caballero et al., 2022). The functions performed by biocrusts exert a beneficial influence on various aspects, including the germination and establishment of seeds, the performance of plants, as well as the population dynamics and behavior of animals (Lan et al., 2015; Couradeau et al., 2016; Weber et al., 2016). Among the ecosystem functions offered by biocrusts, their pivotal role in stabilizing soil and mitigating soil erosion stands as the most vital ecological services in numerous ecoregions (Belnap et al., 2003, 2009; Gao et al., 2017). Munson et al. (2011) documented that biocrusts have the capacity to prevent soil loss caused by wind erosion. Moreover, biocrust also has a significantly inhibitory effect on water erosion. The study conducted by Gao et al. (2017) reported well-developed biocrusts could reduce soil loss by up to 100% in areas experiencing water erosion. Similarly, in the region of semi-arid watersheds, despite large amounts of runoff, the least erosion was observed in areas where biocrusts were present (Rodríguez-Caballero et al., 2014). Furthermore, the magnitude of biocrust coverage is considered the foremost predictor of site stability within local ecosystems (Belnap et al., 2009). Despite the widespread acknowledgment of the soil erosion-reducing capacity of biocrusts, current research on the erosion-resistant mechanisms of biocrusts predominantly centers on arid regions, with virtually no exploration in the realm of tropical coral islands.

In general, the protective impact of biocrusts against erosion is directly related to the extent of their coverage on the soil surface. Biocrusts possess the capacity to shield the erodible surface layer physically or create aggregates through their biomass. The initial aggregation of the biocrust is facilitated by the secretion of extracellular polymeric substances (EPSs) from biocrust organisms. EPSs comprise a combination of polysaccharides, proteins, nucleic acids, lipids, and humic substances, each present in varying proportions and possessing diverse chemical properties and structures (Flemming and Wingender, 2010; Rossi et al., 2018). Indeed, a significant proportion of the organisms found in biocrusts, such as cyanobacteria, green microalgae, and microfungi, possess the ability to produce EPSs that become incorporated within the soil mineral particles and cellular structures (Belnap et al., 2003). Hence, the synthesis of EPSs exhibit a strong correlation with the abundance and diversity of microorganisms (Meng et al., 2006; Zhang et al., 2015). Microbial EPSs could promote the formation of soil aggregates by owing to their viscosity, so that it has a high structural stability (Costa et al., 2018). Belnap et al. (1993) and Bowker et al. (2008) also reported that biocrusts can decrease soil loss mainly due to microbial EPSs bind soil particles smaller than 65 μm together and physically weave them together by filamentous cyanobacteria. Further, the EPSs secreted by the biocrust and the formation of biofilms could relatively increase soil crust thickness with resulting increase in stability against wind force (Kheirfam and Asadzadeh, 2020). Regarding biocrusts, there is a notable variation in microbial abundance and diversity across various biocrust types (Büdel et al., 2009; Weber et al., 2012; Maier et al., 2018; Wang et al., 2022). This variation in microbial composition potentially contributes to the divergence observed in EPSs contents and synthesis. Notably, changes in the environmental conditions (e.g., nutrient elements, contaminants, pH, temperature, and salinity) also had strongly influence in EPSs synthesis (Yang et al., 2018, 2020). Might the variances in environmental conditions engender distinctions in the erosion-resistant mechanisms of biocrusts between arid regions and tropical coral islands?

Meiji Island is a typical coral island in Nansha Archipelagos, South China Sea, and the primary constituents of the soil are coral calcareous sand (Li et al., 2013). Further, Meiji Island highly susceptible to soil erosion due to abundant rainfall, loose coral sand, and extreme environmental conditions, including high salt, alkali, temperature, light, and radiation intensity. Recent years, with the development and utilization of coral islands, the role of biocrusts in reducing soil erosion of coral islands has gradually attracted attention. Despite the widely acknowledged barrier function of biocrusts (Munson et al., 2011; Zhao and Xu, 2013; Gao et al., 2017), the precise mechanism governing the interplay between EPSs concentration, soil erosion resistance, and microbial composition associated with biocrusts remains inadequately comprehended. In this study, our focus lies on examining the alterations induced by biocrusts in the inherent properties of soil that are linked to erodibility, and evaluate the potential impact of biocrusts on soil resistance to erosion throughout the region. Therefore, we aim to address two questions: (1) how biocrust formation affects soil properties, microbial community, and EPSs contents on tropical coral island? (2) does biocrust formation affect the soil erodibility? The outcomes of this study will not only showcase the extent of influence exerted by biocrusts in reducing soil erodibility but also offer a fresh perspective on the ecological restoration of tropical coral islands.

## Materials and methods

### Sampling and storage

**Figure 1:**
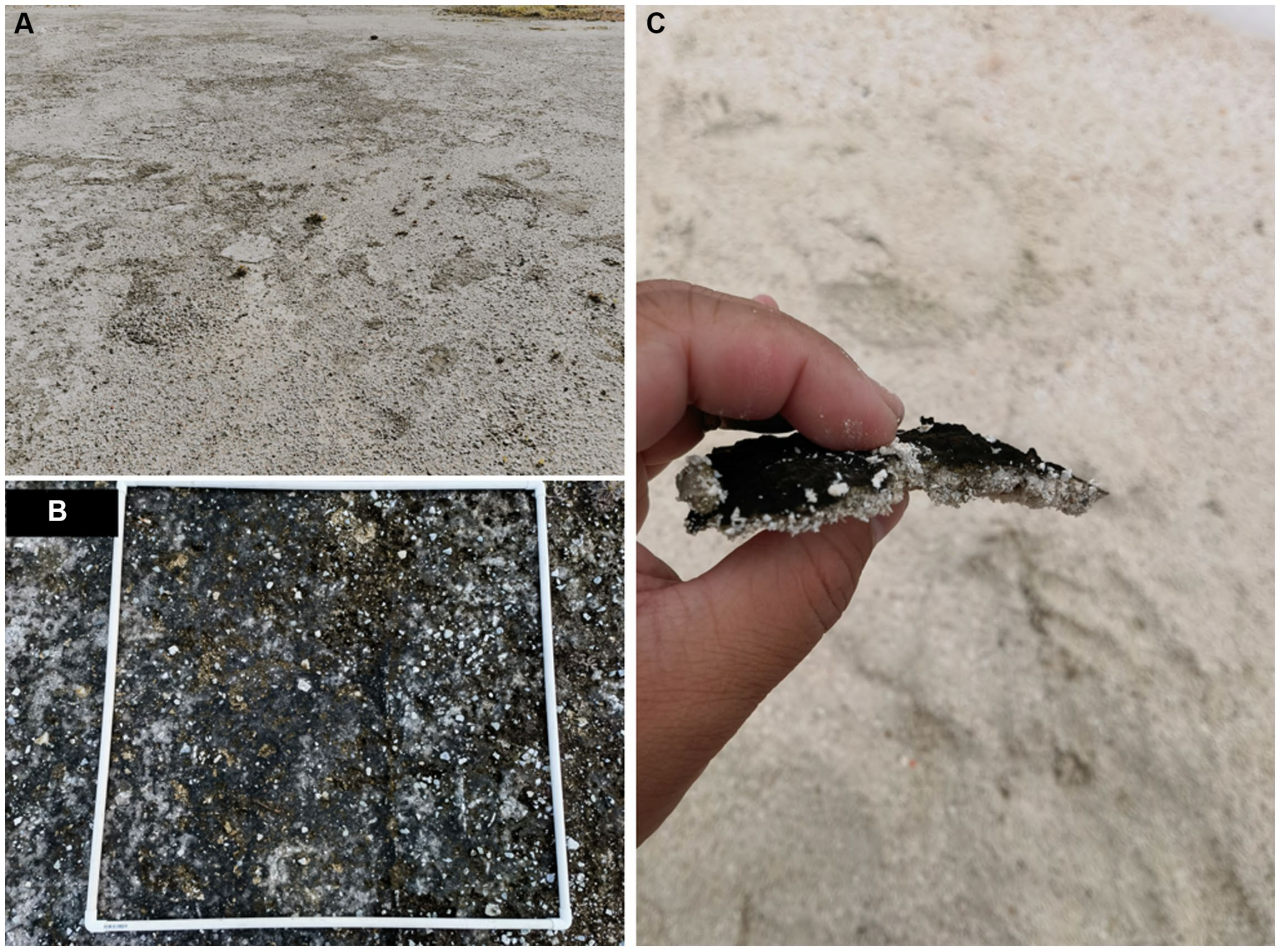
The representative coral land biocrust samples. The landscape view of the study site **(A)**, cyanobacteria-dominated biocrust **(B)**, and the filamentous cyanobacteria associated with sand particles **(C)** were investigated.

The samples of biocrust and bare soil were meticulously gathered from Meiji Island, South China Sea, during October, 2020. This island is influenced by a tropical marine climate, characterized by an average temperature surpassing 27°C and an annual precipitation exceeding 2,000 mm. The coverage of biocrusts on Meiji Island is approximately 6.25%, predominantly composed of cyanobacterium-crust, with almost no presence of lichen-or moss-dominated types (Figure 1; Wang et al., 2022). The surface texture of the biocrust was very similar, all of which were rugose. For per sampling site, precise collection of biocrust (uppermost 0–1 cm layer) and bare soil (uppermost 0–1 cm of adjacent soil lacking evident indications of biocrusts) was performed using sterile equipment. We inserted a sterilized foil sampler into the sampling site and used sterile spatulas and spoons to separate the surface biocrust and bare soil from the underlying soil sample, followed by appropriate preservation methods. In sum, 12 samples were used for sequencing, including 6 bare soil and 6 biocrust samples; a total of 16 samples were used for soil properties testing, including 8 bare soil and 8 biocrust samples. The collection of samples, including biocrusts and bare soil, was carried out with a minimum distance of 100 meters between each sample location.

The samples intended for soil properties assays were meticulously obtained and placed in sealed plastic bags, ensuring a secure environment. These bags were subsequently stored in a temperature-controlled freezer at −20°C. As for the samples designated for DNA extraction, sterile equipment was employed during collection, and the samples were then placed in tubes that containing LifeGuard™ Soil Preservation Solution (MO BIO Laboratories, Carlsbad, CA, United States). These tubes were suitably preserved at a temperature of-80°C until subsequent processing.

### Measurement of the soil physicochemical parameters

The collected samples underwent a process of air-drying at ambient room temperature. Subsequently, they were meticulously sieved through 35 mesh screens to eliminate any coarse impurities. The remaining fine particles were then finely ground using a mortar and pestle. To determine the soil pH, a 1:2.5 (w/w) suspension of the soil samples was prepared, and the pH analysis was conducted using a pH meter (pH 211, Hanna Instruments, Germany), adhering to the method outlined by Acosta-Martínez et al. (2007). Furthermore, the analysis of total organic carbon (TOC) and total nitrogen content (TN) was carried out utilizing an advanced Elemental Analyzer (Flash EA 3000 Thermo Scientific, Milan, Italy), following the methodology established by Zhao et al. (2019). Total phosphorus (TP) was determined by the H_2_SO_4_–HClO_4_ digestion method (Kuo, 1996). Chl *a* content was measured based on described by Wang et al. (2022). The grain-size components of samples were analyzed using a laser particle sizer (Malvern Mastersizer 2000) as previously described (Tian et al., 2019).

### Analysis of EPSs concentrations

EPSs extraction was conducted using a cation exchange resin method with minor modifications, as previously described by Takahashi et al. (2009) and Mai et al. (2022). In summary, artificial seawater with a salinity of 30‰ was added to tubes containing 30 grams of fresh soil sample and mixed in a shaker for 1 h at 4°C in the absence of light. The supernatant was collected after centrifugation at 3,500 rpm at 4°C for 10 min to obtain colloidal EPSs. Subsequently, 20 mL of artificial seawater and 2 grams of activated cation exchange Dowex 50 WX8 resin (in hydrogen form, with a mesh size of 200–400, from Sigma-Aldrich, MO, United States) were added to the remaining soil sample. This mixture was then mixed for 1 h at 4°C in the dark and subsequently centrifuged at 3,500 rpm at 4°C for 10 min to obtain bound EPSs. Both the supernatants of the two EPSs subfractions were purified using dialysis bags (with a 3.5 kDa molecular weight cutoff, incubated at 4°C for 24 h). The purified EPSs samples were then subjected to freeze-drying. Concurrently, the determination of extracellular protein content (EP) and polysaccharide content (EPS) involved the reconstitution of freeze-dried colloidal and bound EPSs powder in 10 mL of deionized water, respectively. The EP concentration was quantified using a modified bicinchoninic acid protein assay kit (Sangon Biotech, Shanghai, China), with bovine serum albumin serving as the standard (Smith et al., 1985). For the evaluation of EPS content, the phenol-sulfuric acid method, as described by DuBois et al. (1956), was employed with glucose as the standard reference. The unit of EPS and EP content is μg/g.

### Estimation of the soil erodibility factor on biocrust

The soil erodibility factor (K) was usually applied for represent soil erodibility (Sharpley and Williams, 1990). This factor has found extensive application in various models utilized for the prediction of soil erosion (Liu et al., 1999; Parysow et al., 2003; Ouyang et al., 2018). The K embodies the inherent susceptibility of soil to undergo denudation and migration when subjected to the erosive forces of raindrop splash and runoff. In total, we collected 16 samples for the determination of the soil erosion coefficient. These samples comprise 8 biocrust and 8 bare soil samples. We employed the foil sampler method for field sampling, followed by laboratory analysis of the collected samples’ soil mechanical composition, which includes the proportion of sand (0.05–2.00 mm, %), silt (0.002–0.05 mm, %), and clay (<0.002 mm, %). Subsequently, we conducted measurements of the organic carbon content for each sample. Finally, we calculated the K (t h MJ^−1^ mm^−1^) for each sample using the formula described by Sharpley and Williams (1990):


K=0.2+0.3exp−0.0256Sa1−Si100∗SiCl+Si0.3∗1−0.25CC+exp3.72−2.95C∗1−0.7SnSn+exp−5.51+22.9Sn

and


Sn=1−Sa100,

where *S_a_* represents the sand content (0.05–2.00 mm, %); *S_i_* reflects the silt content (0.002–0.05 mm, %); *C_l_* reflects the clay content (< 0.002 mm, %); and C is the total organic carbon content (%).

### DNA extraction, amplification, and sequencing

Extraction of total environmental DNA was carried out utilizing DNeasy^®^ PowerSoil^®^ Pro Kit (QIAGEN, United States), followed by monitoring its concentration using a NanoVuePlus Spectrophotometer (GE Healthcare, Little Chalfont, United Kingdom). The primers for amplification of the V4 region of the bacterial 16S rRNA gene, ITS1 region of fungal ITS, and V4-V5 region of archaeal 16S rRNA gene were designed based on Wang et al. (2022). Specifically, the primer of the V4 region of the bacterial 16S rRNA gene was: 515F 5′-GTGCCAGCMGCCGCGGTAA-3′, 806R 5′-GGACTACHV GGGTWTCTAAT-3′; the primer of ITS1 region of fungal ITS was: ITS1f 5′-CTTGGTCATTTAGAGGAAGTAA-3′, ITS2 5′-GCT GCGTTCTTCATCGATGC-3′; and the primer of the V4-V5 region of archaeal 16S rRNA gene was: Arch519F 5′-CAGCCGCCGCGGTAA-3′, Arch915R 5′-GTGCTCCCCCGC CAATTCCT-3′. The products derived from standard thermocycling, involving an annealing temperature of 53°C with 30 cycles for bacterial V4 and archaeal V4-V5 regions, and 53°C with 35 cycles for the ITS1 region, were consolidated and subsequently sequenced on an Illumina Miseq PE300 platform (Majorbio Bio-Pharm Technology, Shanghai, China). The raw sequencing data have been deposited in the National Center for Biotechnology Information.^1^

De-multiplexing raw FASTQ files by applying the in-house perl scripts, filtered by fastp v0.19.6 and merged by FLASH v1.2.7 (Magoč and Salzberg, 2011). The flashed reads were processed using the Quantitative Insights into Microbial Ecology (QIIME) v1.8.0 and UCHIME algorithm-based Gold database to obtain effective tags (Bates et al., 2010; Bokulich et al., 2012). Next, we applied UPARSE 7.1 to cluster the optimized sequences into operational taxonomic units (OTUs) at a 97% sequence similarity level (Edgar, 2013). For each representative sequence, the Silva v138 (for 16S) and Unite 8.0 (for ITS) databases were used to annotate taxonomic information with a confidence threshold of ≥0.5.

### Quantitative PCR

The abundance of bacterial 16S rRNA, fungal ITS, archaeal 16S rRNA, and cyanobacterial 16S rRNA gene were measured by Quantitative PCR (qPCR). The primer used were: for bacteria, 338F (5′- ACTCCTACGGGAGGCAGCAG-3′) and 518R (5′- ATTACCGCGGCTGCTGG-3′) (Maier et al., 2018); for fungi, ITS7 (5′-GTGARTCATCGARTCTTTG-3′) and ITS4 (5’-TCCTCCGCTTATTGATATGC-3′) (Zhang et al., 2018); for archaea, Arch349F (5′-GYGCASCAGKCGMGAAW-3′) and Arch806R (5′-GGACTACVSGGGTATCTAAT-3′) (Zhao et al., 2020); for cyanobacteria, CYA 359F (5′-GGGGAATYTTCCGCAATGGG-3′) and an equimolar mixture of CYA-781RA (5′-GACTACTGGGGTATCTAATCCCATT-3′) and CYA-781RB (5′-GACTACAGGGGTATCTAATCCCTTT-3′) (Nübel et al., 1997), respectively. The qPCR reactions were conducted in triplicate using a CFX96 Real-Time System (Bio-Rad Inc., United States). The thermal profile comprised an initial denaturation step at 95°C for 30 s, succeeded by 40 cycles of 95°C for 10 s, 55°C for 30 s, and 72°C for 30 s. Notably, the assay efficiency for all targeted genes was recorded as 98%, accompanied by a standard curve regression coefficient (R^2^) exceeding 0.995.

### Statistical analyses

A *t*-test was performed to analysis the soil characteristics between biocrust and bare soil, employing SPSS 18 software. Correlations between EPSs contents and the soil erodibility factor K were evaluated with logistic regression. Permutational multivariate analysis of variance determined the statistical significance of differences between biocrusts and bare soils (Vegan v2.5–3 in R). The canonical correlation analysis (CCA) and redundancy analysis (RDA) investigated the impact of soil properties on the microbial composition (Vegan v2.5–3 package in R). The selection principle of RDA or CCA model: first use the abundance matrix data to do DCA (Detrended Correspondence Analysis), and look at the size of the Axis Lengths of gradient in the analysis results. If it is greater than 4.0, CCA is recommended; if it is between 3.0–4.0, both RDA and CCA are acceptable; if it is less than 3.0, RDA is recommended. Monte Carlo permutation tests inferred the forward selection (permutations = 9,999). The heatmap depicted the correlation between microbial relative abundance and environmental factors through Spearman’s rank correlations (Wang et al., 2022).

## Results

### Determination of soil properties

By determining soil properties that serve as indicators of the biocrust formation process, we found that the biocrusts’ pH values were significantly lower, but TOC, TP, TN, and Chl *a* content exhibited significant increases in biocrusts (Supplementary Table S1). With biocrust formation, biocrusts had more fine particles, including clay and silt compared to bares soil, but the opposite result for sand (0.05–2 mm) content (Supplementary Table S2). The gravel ratio has no significantly difference between biocrust and bare soil.

### Microbial diversity and composition

**Figure 2:**
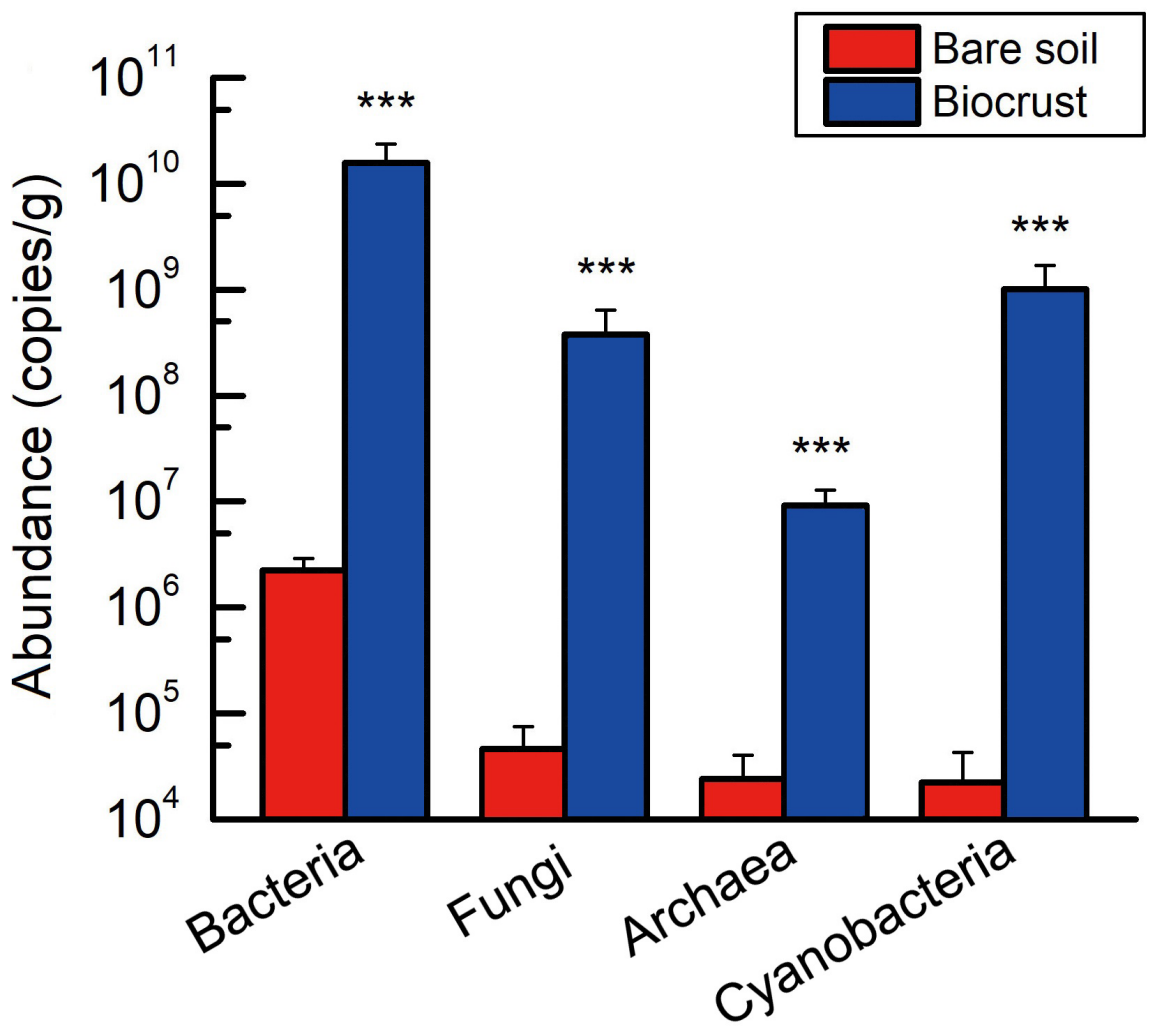
The absolute abundance of bacteria, fungi, archaea, and cyanobacteria within both the bare soil and biocrust samples. All data are presented as Mean ± SD calculated from biological repetition. Significant differences: ***P < 0.001.

Employing qPCR to quantify the abundance of microbiome in bare soil and biocrust, including bacteria, fungi, archaea, and cyanobacteria. The gene copy numbers of these microbial groups were found to be significantly lower in bare soil than that in the biocrusts (Figure 2). Meanwhile, bacterial abundance was significantly higher than that of fungi and archaea in both biocrust and bare soil samples. Moreover, the abundance of cyanobacterial community changed from the lowest to the second highest (Figure 2).

**Figure 3:**
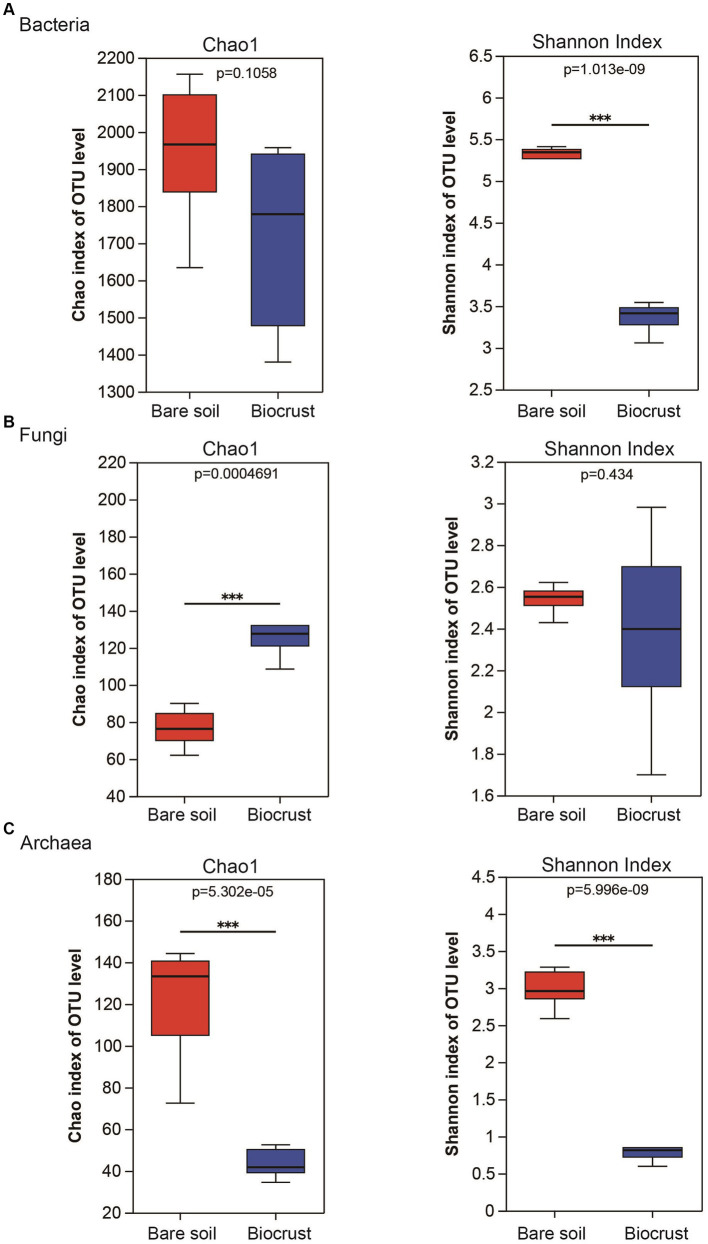
Alteration of richness and diversity of bacterial, fungal and archaeal communities in the biocrust and bare soil samples. The boxplot demonstrates the alpha diversity parameters, Chao1 and Shannon index, across three types of microbial communities, bacteria **(A)**, fungi **(B)**, and archaea **(C)**. Boxes limit the 25th-and 75th percentile with the median presented as line inside. Error bars present the 1st and 99th percentile and outliers are shown as dots below and above. Significant differences: ***P < 0.001.

A comparison of alpha diversity (chao 1 and Shannon index) was conducted across all samples. In the bacterial community, the Chao 1 was no significantly difference between the biocrust and bare soil, while the Shannon index was higher in the bare soil (Figure 3A). In addition, the Chao 1 of the fungal community was lower in the bare soil compared to the biocrusts (Figure 3B). In contrast, the Chao 1 and Shannon index of archaeal community were significantly higher in the bare soil than that in biocrusts (Figure 3C).

**Figure 4:**
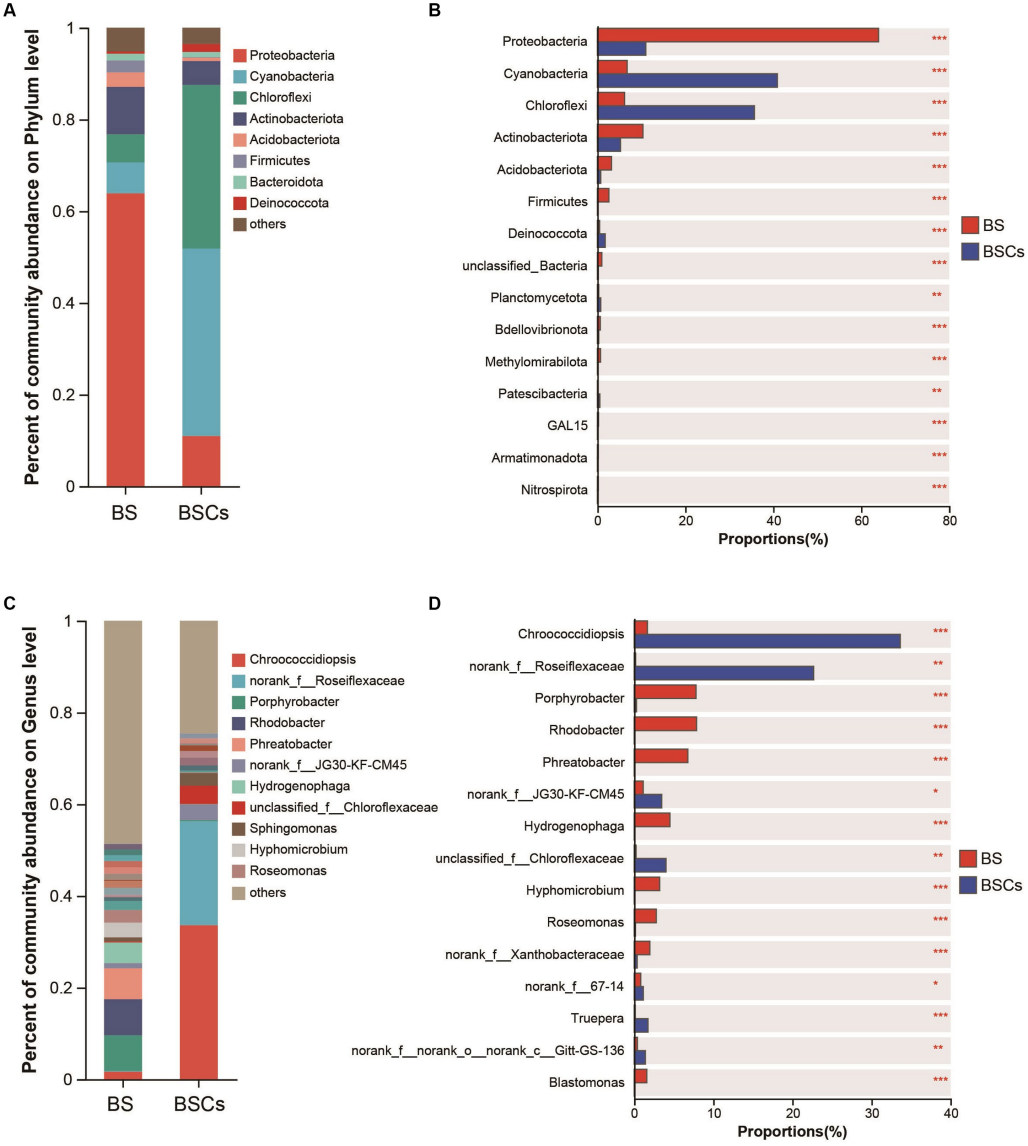
Analysis of differences of microbial composition between bare soil and biocrusts. **(A)** Relative abundance of the major bacterial taxa in biocrust and bare soil; **(B)** difference analysis of dominant bacterial taxa at phylum level; **(C)** relative abundance of the major bacterial taxa in biocrust and bare soil at genus level; **(D)** difference analysis of dominant bacterial taxa at genus level. Based on amplicon 16S rRNA gene data. Significant differences: ***p < 0.001, **p < 0.01, *p < 0.05. BS, bare soil; BSCs, biocrusts.

Within the bacterial community, the most abundant taxa in the biocrust at the phylum level were Cyanobacteria (average relative abundance of 40.90%), followed by Chloroflexi (35.69%), Proteobacteria (10.97%), and Actinobacteriota (5.20%) (Figure 4A). Notably, Cyanobacteria and Chloroflexi were significantly higher in biocrust than that in bare soil (Figure 4B). Among the bare soil samples, phyla Proteobacteria was dominant (63.91%), followed by Actinobacteriota (10.32%), Cyanobacteria (6.73%), and Chloroflexi (6.16%) (Figure 4A). At the genus level, the most prevalent taxa in the biocrust were *Chroococcidiopsis* (33.62%) and *norank_f__Roseiflexaceae* (22.67%) (Figure 4C), both exhibiting a substantial abundance over the bare soil samples (Figure 4D). Conversely, the most abundant genera in the bare soil were *Rhodobacter* (7.87%), *Porphyrobacter* (7.80%), and *Phreatobacter* (6.76%) (Figure 4C). In the fungal community, the most prevalent phylum is Ascomycota (57.84%), yet it displays no significant variance between the biocrust and bare soil (Supplementary Figures S2A,B). However, at the genus level, the biocrust exhibits higher abundance of *unclassified_k__Fungi* (40.94%), *Emericellopsis* (28.01%), and *unclassified_p__Ascomycota* (25.63%), all of which significantly higher compared to bare soil (Supplementary Figures S1C,D). For archaeal community, the most abundant taxa within the biocrust were Crenarchaeota (86.21%) and Halobacterota (9.22%), both significantly higher compared to bare soil (Supplementary Figures S2A,B). The biocrust was dominated by *Candidatus_Nitrocosmicus* (82.22%) at genus level, followed by *Haladaptatus* (8.88%), with both genera substantially surpassing the abundance found in bare soil (Supplementary Figures S3C,D). Based on the 16S rRNA sequencing results, we conducted an annotative analysis of the species composition within the phylum Cyanobacteria. The relative abundance of dominant cyanobacterial taxa significantly increased with biocrust formation. Specifically, the abundance of *Chroococcidiopsis* (80.65%) was significantly higher in biocrust than that in bare soil (Supplementary Figure S4), while *unclassified_o_Thermosynechococcales* (21%), *unclassified_f_Nostocaceae* (11.57%), and *Chlorogloeopsis* (11.11%) were significantly higher in bare soil (Supplementary Figure S4). This portion of the findings aligns with the outcomes obtained through the amplification utilizing cyanobacterial-specific primers. Relevant data is accessible in Supplementary Table S3 (unpublished data).

**Figure 5:**
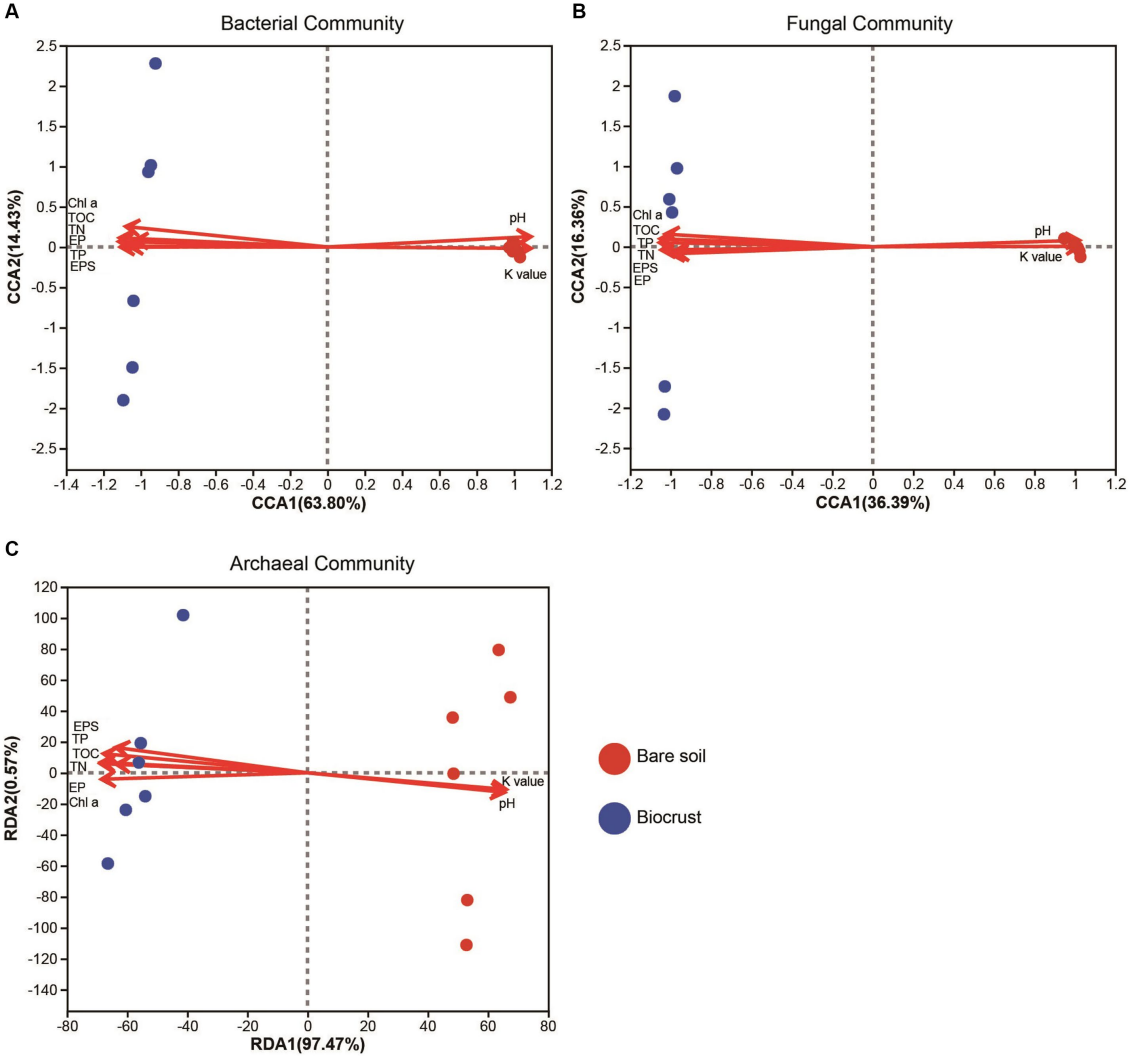
The canonical correlation analysis of bacterial **(A)** and fungal **(B)** community structure and its correlation with environmental factors. The redundancy analysis of archaeal **(C)** community and its correlation with environmental factors. Circle shape represents soil sample. TN, total nitrogen; TOC, total organic carbon; TP, total phosphorus; EP, extracellular protein; EPS, extracellular polysaccharide; Chl a, chlorophyll a; K value, soil erodibility factor.

Through PERMANOVA, we found significant differences in the microbiome between bare soil and the biocrusts, including bacterial, fungal, and archaeal communities (Supplementary Table S3). In addition, the results of CCA and RDA showed that microbial composition and structure (e.g., bacteria, fungi, and archaea) was highly correlated to the soil properties, including Chl *a*, TOC, TN, pH, and TP (Figure 5). Changes in nutrients might have contributed to changes in microbial composition of the biocrusts.

### Measurement of soil erodibility factor

**Figure 6:**
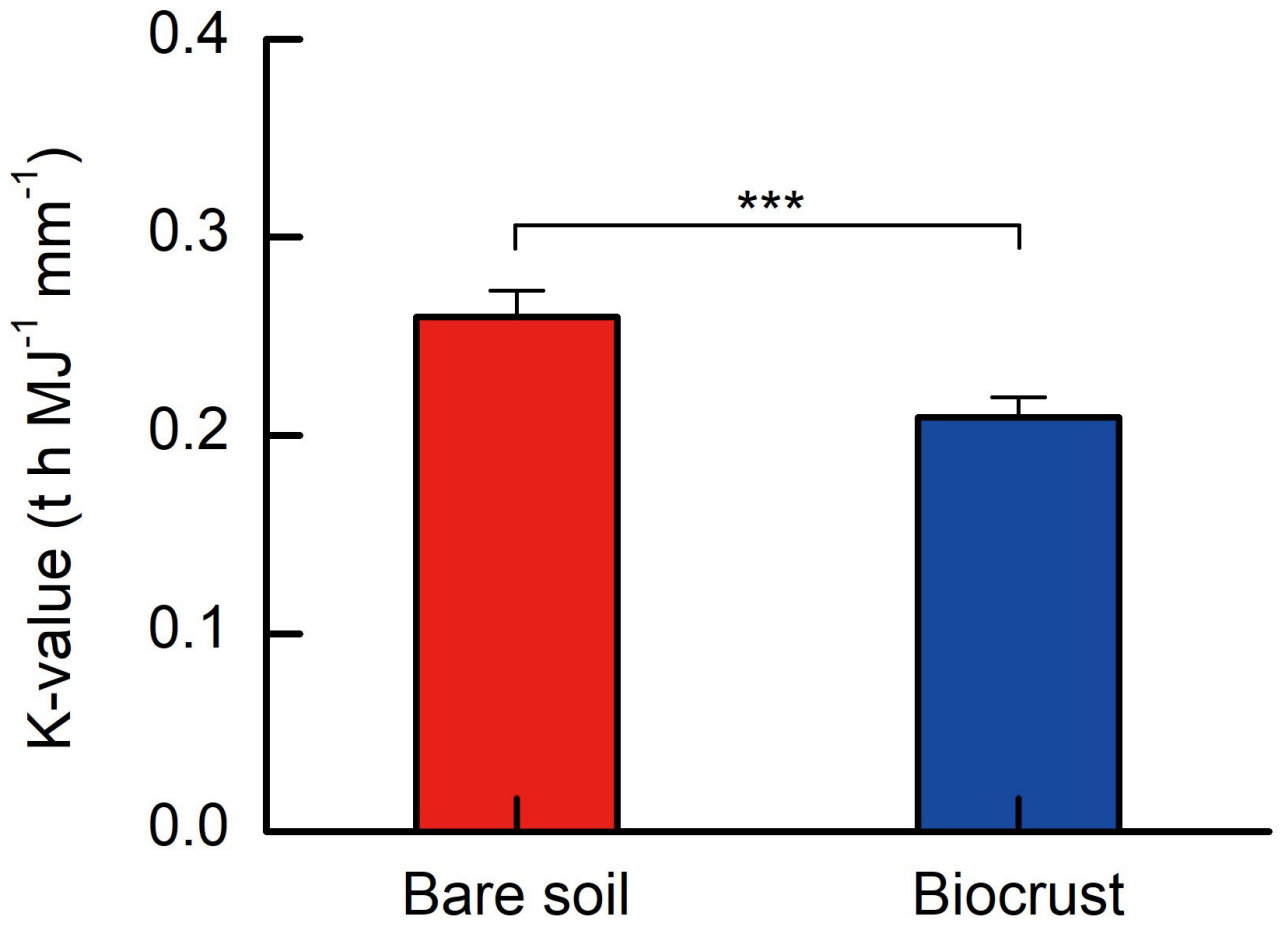
Soil erodibility factor (K) from biocrusts and bare soils. All data are presented as Mean ± SD calculated from biological repetition. Significant differences: ***P < 0.001.

The soil erodibility factor (*K*-value) ranged from 0.264 to 0.280 t h MJ^−1^ mm^−1^, and 0.190 to 0.221 t h MJ^−1^ mm^−1^ on bare soils and biocrusts, respectively. On tropical coral island, the K-value exhibited a notable decrease of approximately 25% in soils influenced by biocrust compared to bare soils, as illustrated in Figure 5. A *t*-test analysis revealed that the K-value in biocrust-influenced soils was significantly lower than that in bare soils (Figure 6).

### Relationship among the soil erodibility factor, EPSs, microbial components, and soil properties

**Figure 7:**
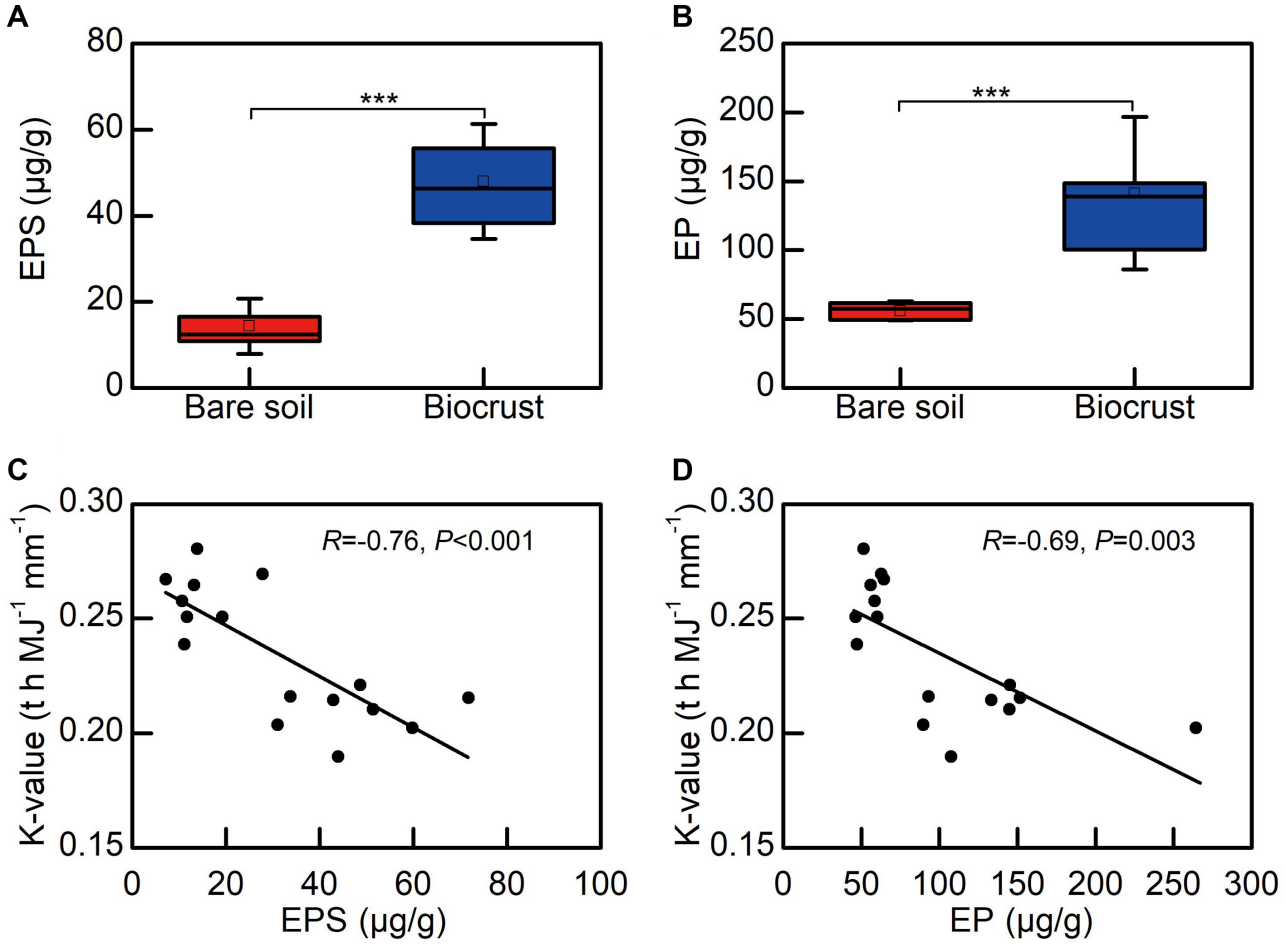
The relationship between EPS and soil erodibility factor (K). **(A)** The content of extracellular polysaccharide, **(B)** the content of extracellular protein, **(C)** the correlation between extracellular polysaccharide and K, **(D)** the correlation between extracellular protein and K. Each circle shape represents different soil sample. EPS, extracellular polysaccharide; EP, extracellular protein.

We observed that EPSs contents, including EP and EPS, in biocrusts were significantly higher than that in bare soil. Notably, both EPS and EP exhibited a significant negative correlation with the soil erodibility factor (Figures 7C,D).

**Figure 8:**
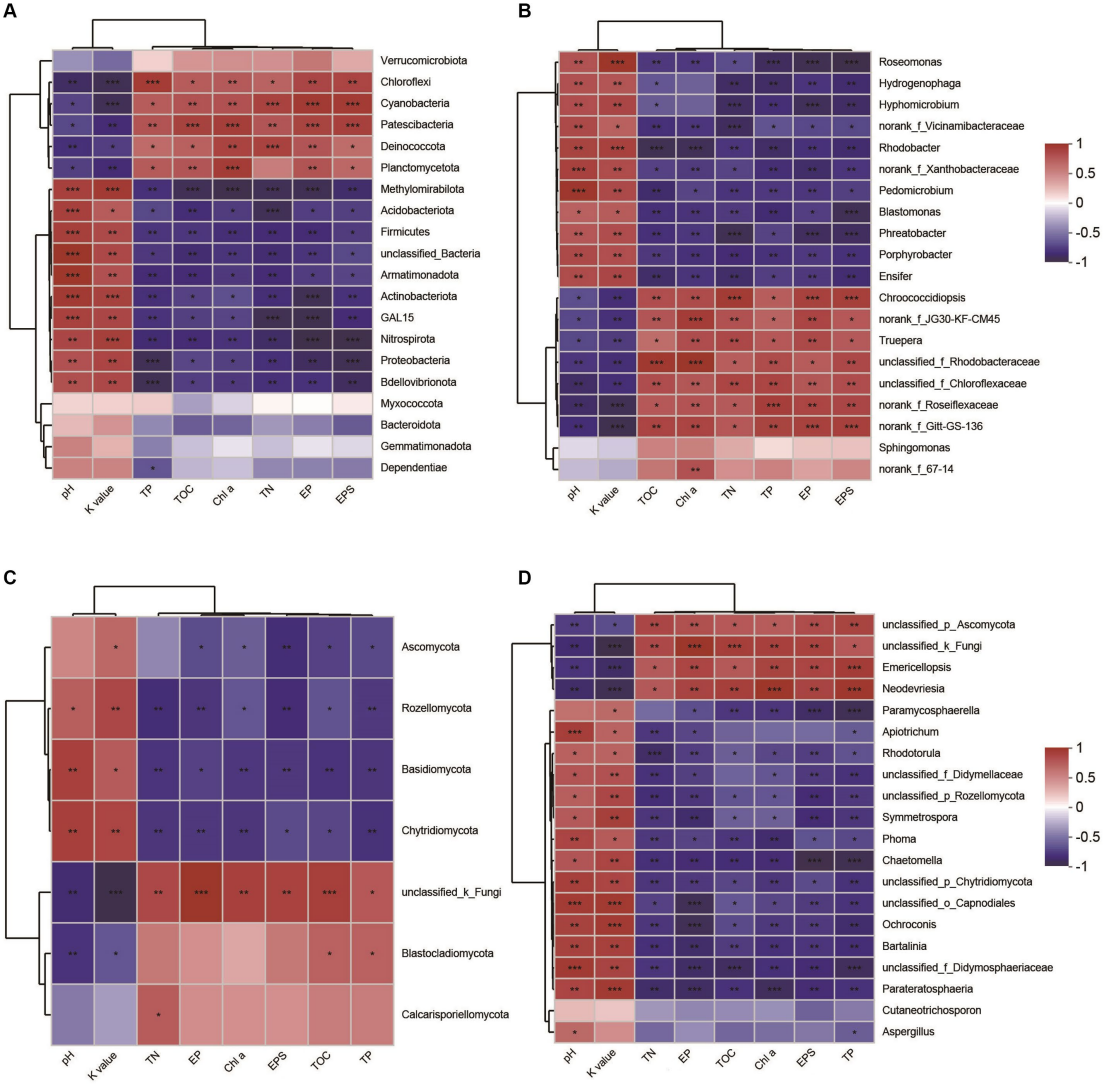
Spearman’s correlation analysis between microbial taxa and soil properties. Bacterial taxa and its correlations with soil parameters **(A)** phylum level, **(B)** genus level. Fungal taxa and tis correlations with soil properties **(C)** phylum level, **(D)** genus level. Red illustrates positive correlations, while blue indicates negative correlations, significant differences: ***p < 0.001, **p < 0.01, *p < 0.05. TN, total nitrogen; TOC, total organic carbon; TP, total phosphorus; EP, extracellular protein; EPS, extracellular polysaccharide; Chl a, chlorophyll a; K value, soil erodibility factor.

Through the analysis of the spearman correlation heatmap, we found that the dominant phyla Proteobacteria, Actinobacteria, Acidobacteriota, Basidiomycota, and Thermoplasmatota were significantly negatively correlated to EPSs content, but the other phyla, including Cyanobacteria, Chloroflexi, Deinococcota, and Crenarchaeota were significantly positively correlated to EPSs contents (Figures 8A,C; Supplementary Figure S5A). At genus level, the dominant taxa, including *Chroococcidiopsis*, *norank_f_Roseiflexaceae*, *Emericellopsis*, and *Candidatus_Nitrocosmicus*, were observed to be significantly positively correlated to EPSs contents (Figures 8B,D; Supplementary Figure S5B). In addition, we also found that these microbial taxa were significantly positively associated with soil properties, including TOC, TN, and Chl a, but not pH and soil erodibility factor (Figure 8; Supplementary Figure S5).

## Discussion

### Biocrust formation enhances EPSs and reduces the soil erodibility factor in the tropical coral island

The study clearly demonstrated that soil erodibility factor was significantly decreased with biocrust formation (Figure 6). Similar protective characteristics (e.g., alleviation of erosion) have likewise been documented for alternative vegetative cover and mangrove ecosystems (Zhou and Shangguan, 2005; Mai et al., 2022). Furthermore, our findings demonstrated a notable negative correlation between the K-value and the abundance of EPSs, including EP and EPS (Figures 7C,D). Consistently, we also observed that EPSs content was rose after biocrust formation (Figures 7A,B). Diminished soil erodibility can be attributed in part to the adhesion of EPS and EP, which, in turn, promote the coalescence of microbial cells and microparticles (Meng et al., 2019). In this investigation, the proportion of silt (0.002–0.05 mm, %) and clay (<0.002 mm, %) were significantly higher in the biocrusts compared to bare soil. This signifies that, under the influence of EPS and EP adsorption, the formation of stable aggregate structures in the biocrusts is rendered more attainable. Simultaneously, EP bolster the architecture of EPSs, thereby facilitating the amalgamation of cells and upholding the steadfastness of biofilms (Flemming et al., 2007; Mai et al., 2022). Both neutral and hydrophobic EPS, on the other hand, heighten the ionic strength and play pivotal roles in the adsorption of EPS (Adav et al., 2008; Sharma and Sarkar, 2013). Therefore, the amplified quantities of EP and EPS engendered by the formation of biocrusts contribute to the promotion of microparticle adsorption and aggregation. Furthermore, the investigation conducted by Flemming and Wingender (2010) further elucidated that EPSs possesses a multitude of binding sites due to its varied functional groups. These binding sites enable the interaction and attachment of macromolecules through forces such as hydrogen bonds and electrostatic interactions, thereby fostering enhanced cohesion (Flemming and Wingender, 2010). Bowker et al. (2008) also reported that the concentration of EPSs could be used as a proxy measure of soil stability in the field. Additionally, the excretion of EPSs by biocrusts contributes to the formation of an exopolymeric matrix, which facilitates the cohesive bonding of bacterial filaments to agglomerated soil particles, as demonstrated by Garcia-Pichel and Wojciechowski (2009). This process establishes a conducive microenvironment for nutrient availability and utilization (Garcia-Pichel and Wojciechowski, 2009). Simultaneously, the exopolymeric matrix, which is an intricate network of EPSs, exhibits varying degrees of attachment to cells and sediments. It can manifest as loosely affiliated material or adopt a more compact configuration, such as tightly bound substances or cyanobacterial sheaths (Rossi et al., 2018). This elaborate framework contributes to enhanced soil stability and fortifies resistance against erosion, as emphasized by Rossi et al. (2018). In the current investigation, we have noted that the EPSs generated by biocrusts possess the capacity to exert influence over the adsorption and aggregation of soil microparticles. Consequently, this phenomenon facilitates the promotion and sustenance of soil macro-aggregate formation while concurrently bolstering the overall stability of the soil structure. Ultimately, these mechanisms culminate in the enhancement of soil’s resistance against erosion.

In arid desert regions, the adhesion mechanisms of biocrusts are widely acknowledged. During their initial developmental stages, certain bacterial constituents of biocrusts, such as Cyanobacteria and Chloroflexi, have been observed to foster soil aggregate formation through the production of EPSs (Mager and Thomas, 2011; Cania et al., 2020). These EPSs served as the “adhesive agents” binding soil particles together. Furthermore, the filamentous structures and EPSs produced by cyanobacteria can create intricate network structures (Zhang, 2005). This network not only binds mineral particles and finer particles but also captures them on the filamentous surfaces, thereby promoting the formation and development of biocrusts (Zhang, 2005). In this study, as biocrusts formed, there was a significant increase in the relative abundance of Cyanobacteria and Chloroflexi, along with a noticeable elevation in EPSs content. Consequently, we hypothesize that the adhesion mechanism of tropical coral island biocrusts shares similarities with those in arid desert areas. However, in this investigation, we observed a significant decrease in pH values as biocrusts formed, which contrasts with conditions in arid regions (Maier et al., 2018; Ghiloufi et al., 2019). The substantial pH reduction suggests an abundance of H^+^ ions within the EPSs secreted by island biocrusts. Given that the calcium carbonate proportion in coral calcareous sand reaches as high as 95%, these H^+^ ions can alter the ion balance of carbonate salts in the soil, thereby promoting mineral precipitation (Zheng and Qian, 2020). This, in turn, leads to an increase in soil particle size. Furthermore, the presence of carbonate salt precipitation in the soil enhances the interparticle adhesion, creating a consolidation effect akin to what is observed in the process of cement solidification (Al-Salloum et al., 2017). This disparity represents the distinguishing feature between the adhesion process of tropical island biocrusts and traditional biocrusts.

Furthermore, EPSs could also create a nutrient-rich microenvironment by capturing airborne silt and clay particles through exploiting its adhesion (Souza-Egipsy et al., 2004). Prior research has extensively documented that the efficacy of biocrusts in capturing dust particles exhibits a progressive increase during the maturation process of these intricate ecosystems (Williams et al., 2012). Notably, among the diverse biocrust types, moss-lichen pinnacled crusts have been found to possess the highest degree of dust capture efficiency and exhibit significant potential in this regard (Williams et al., 2012). Furthermore, apart from their ability to ensnare airborne dust particles, EPSs also have the capacity to accumulate various nutrients and molecules. The incorporation of extracellular enzymes within the EPSs matrix facilitates the establishment of an extracellular digestive system, wherein compounds from the aqueous phase are captured and utilized as nourishing elements and sources of energy (Flemming and Wingender, 2010). Concurrently, a series of studies has provided compelling evidence regarding the remarkable capacity of EPSs to adsorb metal ions, thereby contributing to the remediation of heavy metal contamination and the restoration of polluted environments (Gadd, 2010; Costa et al., 2018).

However, the anti-erosion mechanism of biocrusts is very complex. In addition to EPSs, hydrodynamics, topography, soil particle distribution, and nutrient status may also affect soil erodibility (Gao et al., 2017; Yang et al., 2022). Undertaking more comprehensive investigations that specifically delve into the role of biocrust formation in mitigating run-off and preventing erosion would undoubtedly be a valuable pursuit.

### Potential microbial taxa contributing to EPSs production and erosion resistance

In general, alterations in microbial composition and structure wield a pivotal influence over the secretion and production of EPSs. Enhanced nutrient content and elevated microbial abundance could promote protein and polysaccharide synthesis, result in increased EPSs accumulation in biocrusts (Mager and Thomas, 2011). Lan et al. (2022) reported that different microorganisms have different EPSs secretion capabilities, implying that shifts in microbial composition and structure could consequently induce alterations in EPSs content within biocrusts. In this study, our findings demonstrated that microbial abundance, including bacteria, fungi, archaea, and cyanobacteria, was significantly increased with biocrust formation (Figure 2), which is in line with the results of the study conducted at the Colorado Plateau and Succulent Karoo (Garcia-Pichel et al., 2003; Maier et al., 2018). Moreover, we conducted an analysis of environmental drivers affecting microbial composition in the coral islands. The CCA and RDA showed that microbial composition was significantly correlated to Chl *a* contents, TOC, TP, and TN (Figure 5). As important nutrients for the development of biocrusts, TOC, TP, and TN have been indicated to shape the microbial composition of biocrusts (Schulz et al., 2016; Zhang et al., 2016). Our results indicated that phylum Cyanobacteria, Chloroflexi, and Crenarchaeota might be essential and effective in EPSs secretion through differential analysis at the phylum level (Figure 8; Supplementary Figure S3). Notably, the relative abundances of biocrusts’ major microbial phyla were significantly increased with biocrust formation (Figure 8; Supplementary Figure S3), which similar to our recent publication (Wang et al., 2022). Thus, we speculated that these three major microbial phyla were the main contributors. Specifically, cyanobacteria were the most important microbial phyla in the cyanobacterium-biocrust (Belnap et al., 2016), which produced EPSs that increase soil carbon pools as carbohydrates through CO_2_ fixation. Moreover, the EPSs secreted by cyanobacteria serve as architectural elements, enveloping bacterial cells and intermingling with soil particles. This collaborative interplay results in the formation of heterogeneous clumps or aggregates within the topsoil (Mager and Thomas, 2011). Besides, filamentous cyanobacteria had a greater ability to entangle surface soil particles, as they could reach between soil particles or into deeper biocrusts, facilitated the formation of aggregates and maintained the stability, so that decreased carbon loss from erosion (Mager and Thomas, 2011). Therefore, cyanobacteria, especially filamentous cyanobacterial EPSs, play a key role in stabilizing coral calcareous sand, and preventing the adverse consequences of erosion (such as nutrient and organic matter loss, and dust production). Phototrophic filamentous Chloroflexi thrive in compatible anaerobic environments (Xian et al., 2020), are acknowledged for their copious production of EPSs within their habitat, and possess the capability to fix CO_2_ through a multitude of avenues, encompassing the Calvin cycle, Arnon–Buchanan cycle, and 3-hydroxypropionate bicycle (Ma et al., 2019; Xian et al., 2020). The EPSs secreted by Chloroflexi have the propensity to stimulate the aggregation of soil particles and the formation of soil aggregates, thus effectively mitigating soil erosion (Mager and Thomas, 2011). Further, the dominant archaeal phylum, Crenarchaeota, exhibits high enrichment within biocrusts and plays a significant role as a biogeochemical catalyst in the global nitrogen cycling (Nicol and Schleper, 2006). Meanwhile, the protein or glycoprotein subunits were found in the Crenarchaeota surface layer (Huber and Stetter, 2001). Significantly, the filamentous structure and the secretion of EPSs are shared attributes among the phyla Cyanobacteria and Chloroflexi. These characteristics assume a crucial role in both the formation and the preservation of soil aggregates, thereby contributing to the overall stability of the soil structure. Although all these microorganisms have the capacity to produce EPSs, we are not certain about the primary source of EPSs in this study. Based on the qPCR results, we observed that the abundances of bacteria and cyanobacteria were significantly higher than that of fungi and archaea, leading us to speculate that bacteria are the major contributors to EPSs content in this study. Prior research has indicated that cyanobacteria prefer alkaline environments (Belnap et al., 2003), while Chloroflexi thrive in acidic conditions (Jones et al., 2009; Santofimia et al., 2013; Cania et al., 2020). This accounts for the significantly higher relative abundance of cyanobacteria in the biocrusts of this study, as the alkaline soil environment of the coral island is more conducive to their growth, facilitating greater EPSs production. Therefore, we hypothesize that EPSs primarily originates from cyanobacteria. The amount of EPSs produced by bacterial communities in natural environments can vary depending on soil types (Hu et al., 2002). Moreover, the biochemical composition of EPSs is not specific to particular bacterial groups but rather controlled by environmental conditions and nutrient levels (Nicolaus et al., 1999). Thus, predicting the quantity of EPSs produced by soil microorganisms and tracing its sources presents a challenge. Nevertheless, in the initial stages of biocrust development, EPSs production is predominantly associated with the dominant members of the bacterial community within the biocrust (Cania et al., 2020). In addition, Genus *Chroococcidiopsis* belonged to Phylum Cyanobacteria, is widely described for its ability to metabolize carbon and nitrogen and to secrete polysaccharide and scytonemin (Dineshbabu et al., 2019; Assunção et al., 2021). Genus *Emericellopsis* belongs to Phylum Ascomycota and possesses the myceliar structure. This organism exhibits the capacity to generate substantial quantities of EPSs, thereby offering crucial assistance in enhancing the stability of soil aggregates (Elíades et al., 2006; Gonçalves et al., 2020; Agrawal and Saha, 2022). In our results, the abundance of *Chroococcidiopsis* and *Emericellopsis* in biocrusts was higher, coinciding with increased EPSs concentrations. Nevertheless, within the microbial communities of biocrusts in arid regions, the predominant taxa of cyanobacteria are filamentous or sheath-forming species, such as *Microcoleus vaginatus* and *M. steenstrupii*, contrary to the findings in this study (Mager and Thomas, 2011; Fernandes et al., 2018; Couradeau et al., 2019). This implies a divergence in the assembly mechanisms of biocrusts between tropical coral islands and arid regions. The exploration of these distinct assembly modes will be a focal point of our future investigations, bearing crucial scientific significance in elucidating the formation patterns of tropical coral island biocrusts.

## Conclusion

In this study, we explore the anti-erosion role of biocrusts on tropical coral islands from the perspective of microbial EPSs. We found that after biocrust formation, soil erodibility factor was significantly reduced, whereas EPSs contents, specifically, extracellular protein and polysaccharides were significantly increased, demonstrating a significant negative correlation. Coinciding with an increased EPSs level, we reported increased bacterial, fungal, archaeal, and cyanobacterial abundance. Correlation analysis documented that Cyanobacteria, Chloroflexi, Deinococcota, and Crenarchaeota as potential microbials promoting EPSs and reducing soil erosion. In summation, this study furnishes substantiation of the integral function of biocrusts in averting soil erosion, while elucidating the contribution of microbial EPSs in the amelioration of soil erosion. Moreover, the evidence establishes a foundation for biocrust-directed environmental restoration on tropical coral islands, South China Sea.

## Data availability statement

The datasets presented in this study can be found in online repositories. The names of the repository/repositories and accession number(s) can be found at: https://www.ncbi.nlm.nih.gov/, PRJNA1008893.

## Author contributions

LW: Writing – original draft, Writing – review & editing. YH: Writing – original draft, Writing – review & editing. QY: Writing – original draft, Writing – review & editing. ZM: Writing – review & editing. FX: Writing – review & editing LL: Writing – review & editing. SZ: Writing – original draft, Writing – review & editing. JL: Writing – original draft, Writing – review & editing.

## Footnotes

^1^ http://www.ncbi.nlm.nih.gov/bioproject/1008893
