# Supplementary material for: Biocrust reduces the soil erodibility of coral calcareous sand by regulating microbial community and extracellular polymeric substances on tropical coral island, South China Sea
Source: Front Microbiol. 2023 Dec 13;14:1283073. doi: 10.3389/fmicb.2023.1283073 (PMC10751374; doi:10.3389/fmicb.2023.1283073)
Supplement: Supplementary file 1 [file Data_Sheet_1.pdf]

Table S1. Alteration of the different physicochemical and biological parameters in the biocrust and bare soil samples. Mean ± SD, and different letters indicate significant difference among group at $P < 0.05$.

	TOC (%)	TN (%)	pH	TP (mg/g)	Chl a (μg/g)
Bare soil	0.020 ± 0.05 a	0.002 ± 0.001 a	9.63 ± 0.06 a	0.65 ± 0.10 a	0.05 ± 0.02 a
Biocrust	2.252 ± 0.409 b	0.247 ± 0.033 b	8.67 ± 0.28 b	1.43 ± 0.08 b	15.99 ± 4.37 b

Abbreviations: total organic carbon (TOC), total nitrogen (TN), total phosphorus (TP), Chlorophyll *a* (Chl *a*).

Table S2. Soil mechanical composition. Mean ± SD, and different letters indicate significant difference among group at $P < 0.05$.

	<0.002mm	0.002-0.05mm	0.05-2mm	>2mm
Biocrust	0.79±0.33 a	12.68±3.03 a	57.81±4.20 a	28.74±5.03 a
Bare soil	0.08±0.10 b	9.66±2.47 b	63.97±6.19 b	26.29±6.20 b

Table S3. Cyanobacterial community composition obtained by cyanobacterial-specific primers

Genus	Bare soil	Biocrust-A	Biocrust-B	Biocrust-C	Biocrust-D
g__Anabaenopsis	0.001648	0.00050981	0.0017249	0.01086753	0.00503016
g__Chlorogloeopsis	0.153624	0.01822585	0.0305548	0.03824454	0.05395531
g__Chroococcidiopsis	0.253692	0.7657575	0.7086498	0.60291444	0.65524683
g__Cyanosarcina	0.001054	0.0139859	0.0197893	0.01375648	0.03555952
g__Dolichospermum	0.015787	0.00016994	0.0004673	0.00103662	0.00044184
g__Gloeocapsa	0.008361	0.00564194	0.0138075	0.00510664	0.00707792
g__Komarkovaea	0.000535	0.02266973	0.0170023	0.01634803	0.00280398
g__Leptolyngbya	0.001708	0.08671935	0.0448296	0.0539808	0.0194409
g__Nostoc	0.067338	0.00887926	0.0331804	0.019169	0.0542527
g__Pleurocapsa	0.015838	0.02331549	0.0248364	0.04193219	0.03258561
g__Pseudanabaena	0	0.00362818	0.0078511	0.00448636	0.01077407
g__Scytonema	0.004622	0.00077322	0.0033903	0.00896423	0.00650862
g__Stanieria	0.000697	0.00121506	0.0035857	0.02043504	0.0010876
others	0.475096	0.04850879	0.0903305	0.16275809	0.11523494

Table S4. PERMANOVA analysis of microbial community composition between bare soil and biocrust.

Type	F. Model	R^2	P. value	P.adjust
**Bacterial community**				
Type	F. Model	R^2	P. value	P.adjust
BS vs BSCs	55.1433	0.84649	0.003	0.003
**Fungal community**				
Type	F. Model	R^2	P. value	P.adjust
BS vs BSCs	20.34573	0.67046	0.003	0.003
**Archaeal community**				
Type	F. Model	R^2	P. value	P.adjust
BS vs BSCs	68.52063	0.87264	0.003	0.003

Abbreviations: bare soil (BS), biocrusts (BSCs).

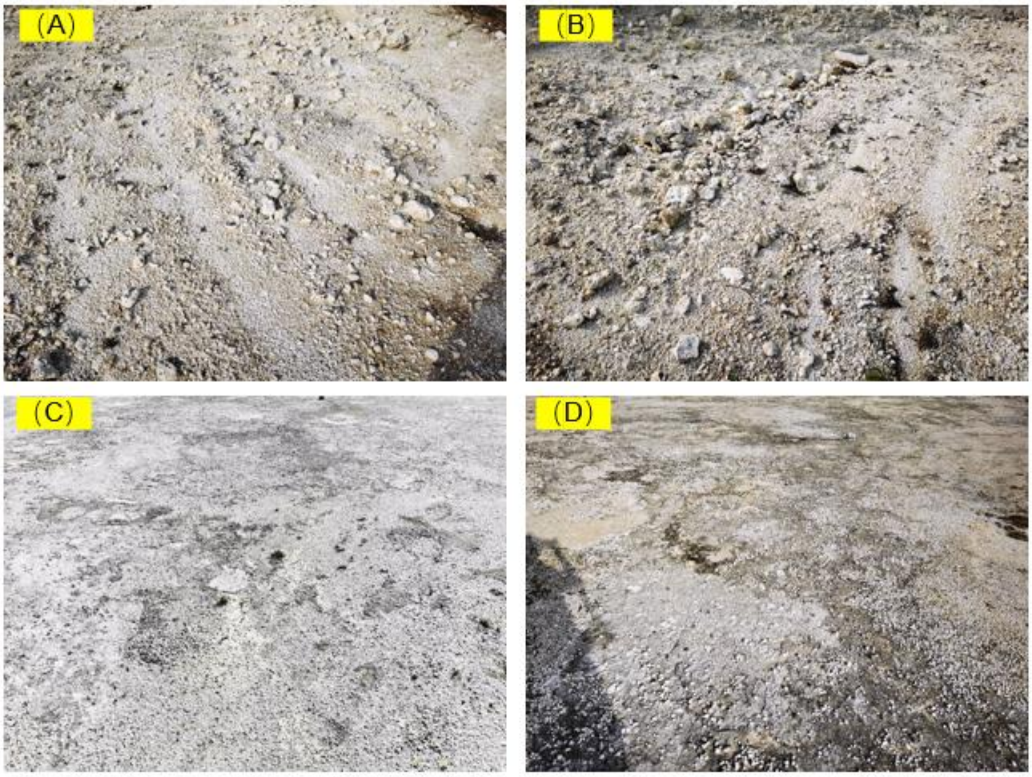

Figure S1 The representative pictures of tropical coral island. (A&B) Sandy areas with low biocrust coverage, (C&D) sandy areas with high biocrust coverage.

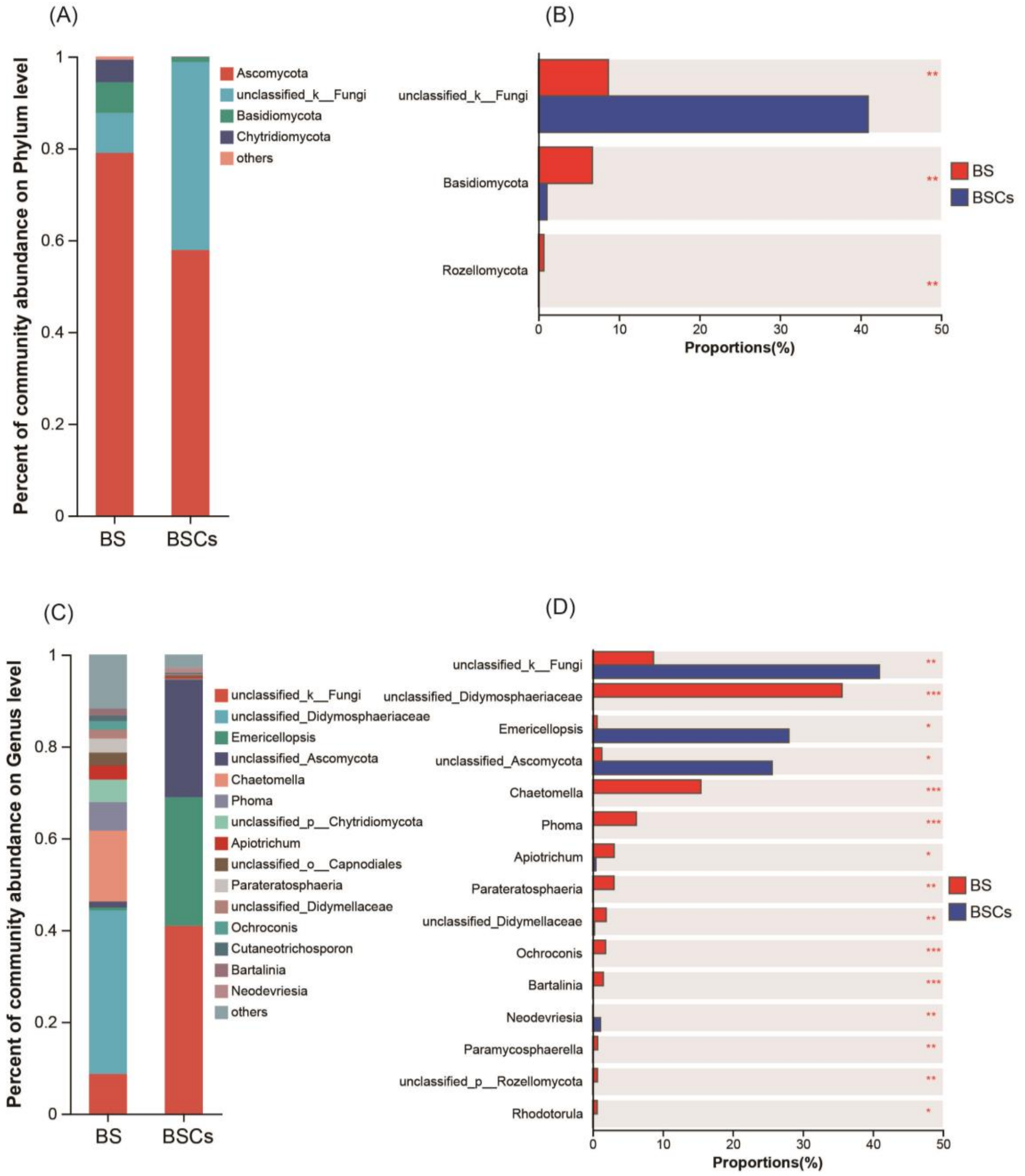

Figure S2 Analysis of differences of fungal composition between bare soil and biocrusts. (A) Relative abundance of the major fungal taxa; (B) difference analysis of dominant fungal taxa at phylum level; (C) relative abundance of the major fungal taxa at genus level; (D) difference analysis of dominant fungal taxa at genus level. Significant differences: ****P*<0.001, ***P*<0.01, **P*<0.05. Abbreviations: bare soil (BS) and biocrusts (BSCs).

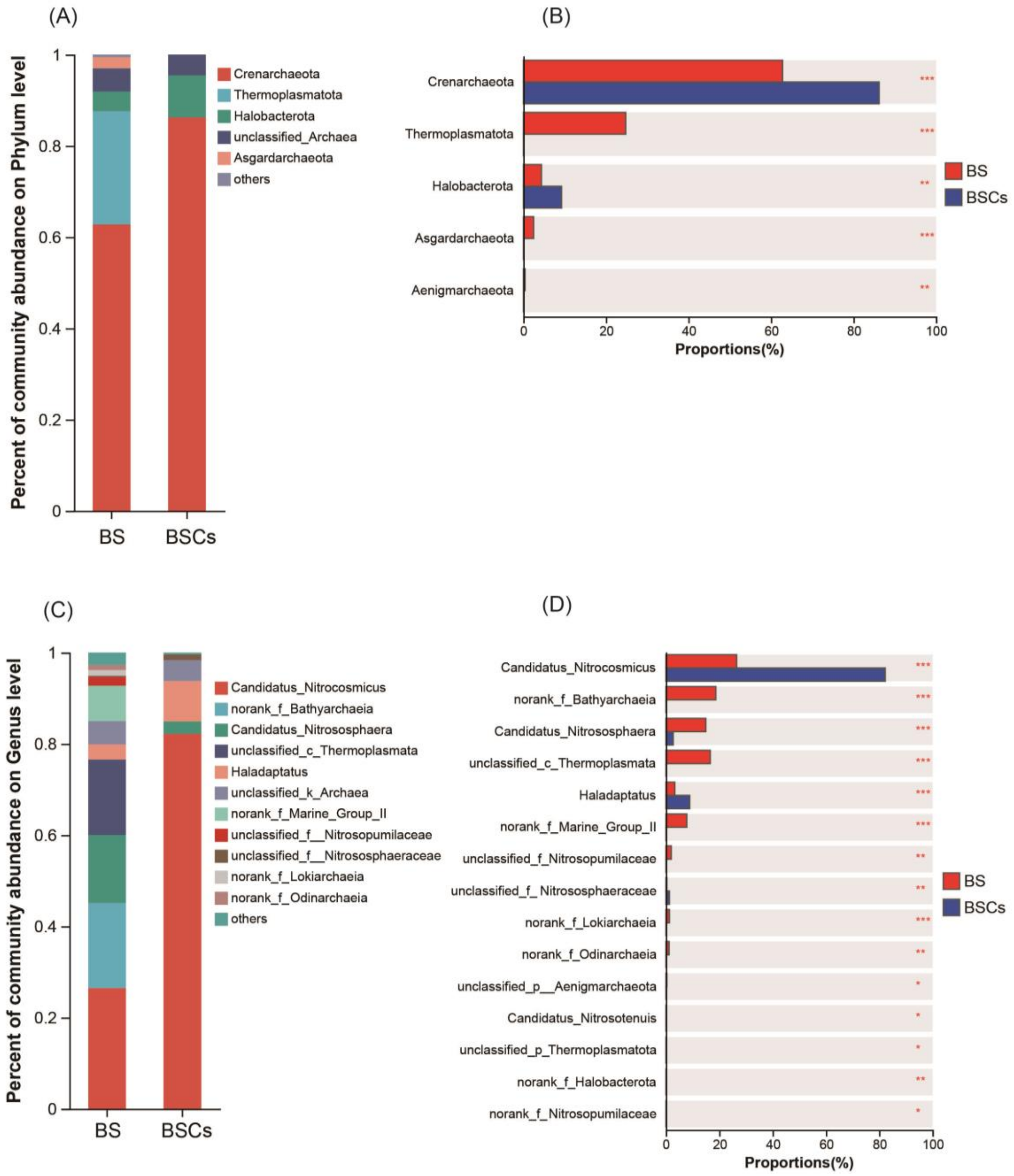

Figure S3 Analysis of differences of archaeal composition between bare soil and biocrusts. (A) Relative abundance of the major archaeal taxa; (B) difference analysis of dominant archaeal taxa at phylum level; (C) relative abundance of the major archaeal taxa at genus level; (D) difference analysis of dominant archaeal taxa at genus level. Significant differences: ***P<0.001, **P<0.01, *P<0.05. Abbreviations: bare soil (BS) and biocrusts (BSCs).

(A)

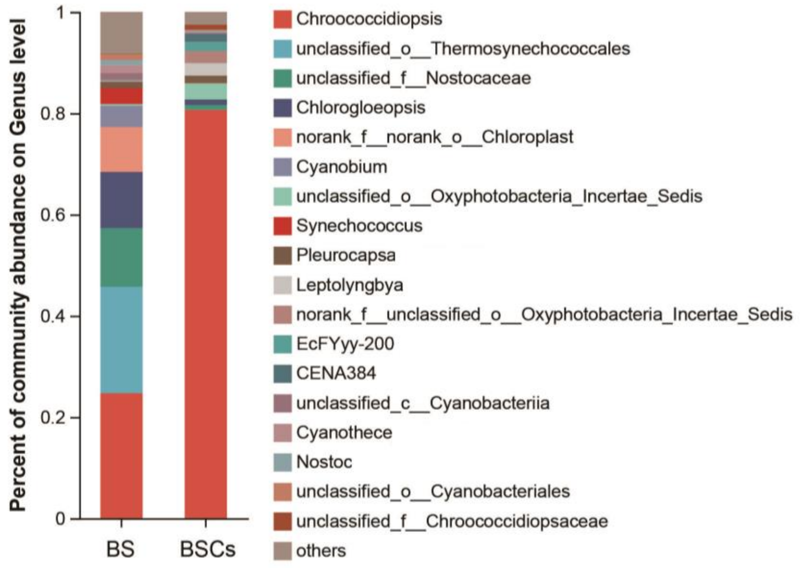

(B)

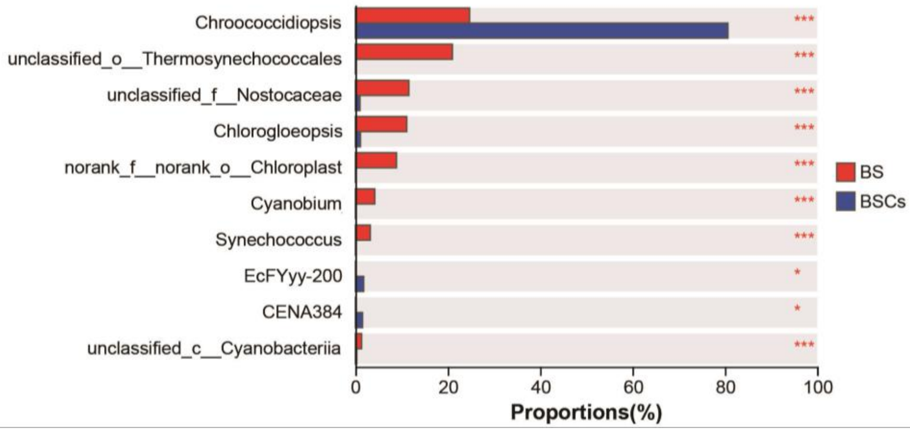

Figure S4 Analysis of differences of cyanobacterial composition between bare soil and biocrusts. (A) Relative abundance of the major cyanobacterial taxa at genus level; (B) difference analysis of dominant cyanobacterial taxa at genus level. Significant differences: ***P<0.001, **P<0.01, *P<0.05. Abbreviations: bare soil (BS) and biocrusts (BSCs).

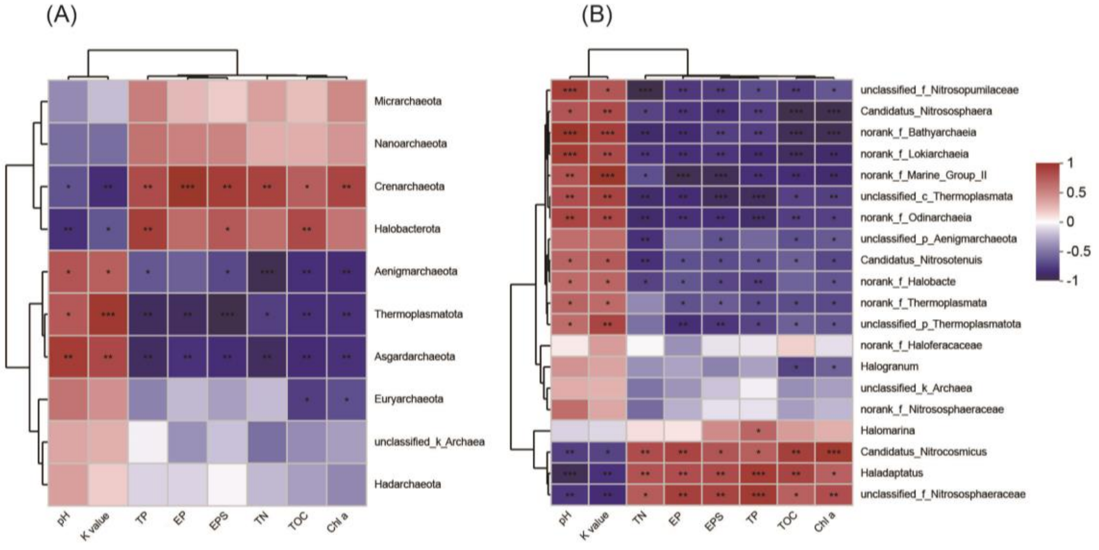

Figure S5 Spearman's correlation analysis between archaeal taxa and soil properties. Archaeal taxa and its correlations with soil parameters (A) phylum level, (B) genus level. Red illustrates positive correlations, while blue indicates negative correlations, significant differences: ***P<0.001, **P<0.01, *P<0.05. Abbreviations: total nitrogen (TN); total organic carbon (TOC); total phosphorus (TP); extracellular protein (EP); extracellular polysaccharide (EPS); chlorophyll *a* (Chl *a*); soil erodibility factor (K value).
